# Clinicopathological Behavior and Oncological Outcomes of Malignant Parotid Tumors in a Pakistani Population

**DOI:** 10.7759/cureus.2157

**Published:** 2018-02-05

**Authors:** Muhammad Faisal, Taskheer Abbas, Mohammad Adeel, Usman Khaleeq, Abdul Wahid Anwer, Kashif Malik, Raza Hussain, Arif Jamshed

**Affiliations:** 1 Department of Surgical Oncology, Shaukat Khanum Memorial Cancer Hospital and Research Center, Lahore, Pakistan; 2 Radiation Oncology, Shaukat Khanum Memorial Cancer Hospital and Research Center, Lahore, Pakistan

**Keywords:** salivary gland neoplasms, malignant parotid tumors, survival outcome

## Abstract

Introduction

The incidence of salivary gland tumors is influenced by geographical and racial factors resulting in diverse histology.  While salivary gland tumors account for a low proportion of head and neck cancers, most malignant tumors of the salivary gland are located in the parotid gland. The goals of this study are to describe the clinicopathological behavior of malignant parotid tumors and explore oncological outcomes related to survival in our Pakistani tertiary care cancer hospital.

Methods

We conducted a retrospective analysis of 209 patients diagnosed with malignant parotid tumors from 2004 to 2016. Data such as demographics, age, gender, histology, grade, clinical and pathological stage, surgical treatment types and adjuvant modalities used were analyzed using SPSS software version 20. We used Kaplan Meier curves to analyze survival data.

Results

The median patient age at diagnosis was 40 years, and the ratio of men to women was 1.2:1. Mucoepidermoid carcinoma was the most common histological variant (with a 50% incidence rate) followed by adenoid cystic carcinoma (13%), and adenocarcinoma (10%). Histology has further categorized these malignant tumors into low (34%), intermediate (28%), and high (21% ) grades. The American Joint Committee on Cancer, seventh edition, clinical staging was Stage I (21%), II (28%), III (15%), and IV (34%). The 5-year survival was 68%, and the 10-year survival was 45%.

Conclusion

Mucoepidermoid carcinoma is the most common malignant parotid histology in our patient population. Advanced age, increased T stage (size > 4 cm), high-grade histology, and cervical nodal involvement decrease overall survival. Open biopsies, piecemeal excisions, and delayed presentation for radiotherapy post-surgery may also have role in adverse outcomes in these malignancies.

## Introduction

Malignant tumors of the parotid gland are characterized by their low incidence rate (1% to 3% of all head and neck cancers) and marked histopathological heterogeneity [1]. Approximately 70% of the malignant tumors of the major salivary glands are in the parotid gland [2]. Tumors in the submandibular and parotid regions are not quite as accessible. For these tumors, fine-needle aspiration (FNA) is the preferred method of biopsy. Several studies have now demonstrated that FNA is a reliable technique achieving a diagnostic result in 88% to 98% of biopsies [3-5]. Superficial parotidectomy preserving the facial nerve has remained the recommended treatment modality, while adjuvant treatment and cervical lymph node removal are usually reserved for advanced stage, high-grade histology tumors [6-8]. Factors influencing the oncological outcome of these tumors include the level of histological diversity, primary tumor status, regional nodal involvement, and adjuvant use of chemo-radiotherapy. Our study aims to describe the clinicopathological behavior of malignant parotid tumors and explore the oncological outcomes related to survival from a tertiary care cancer hospital in Pakistan.

## Materials and methods

We conducted a retrospective analysis of 209 patients with a malignant parotid tumor who received treatment from January 2004 to December 2014 at Shaukat Khanum Memorial Cancer Hospital and Research Center in Lahore, Pakistan.

All patients presented to the head and neck clinic after histological confirmation by FNA cytology. Imaging such as computerized tomography or magnetic resonance imaging was utilized to assess the local extent of the disease as well as the nodal status.

A head and neck multi-disciplinary team discussed each patient before making a final decision on treatment.

Surgical procedures performed were superficial parotidectomy (nerve sparing), completion parotidectomy (operation following previous incomplete procedure), radical parotidectomy (nerve sacrificing), extended radical parotidectomy (sacrificing adjacent muscles and soft tissue such as skin depending upon local extension of the disease). Neck dissection as part of the procedure was offered to those with tumor size of >3 cm, clinically involved lymph nodes, nodes found to be positive during intraoperative frozen sections, and high-grade malignancy.

Similarly, adjuvant radiotherapy was offered to those with high-grade malignancy, close or positive resection margins, and tumor size >4 cm, and positive lymph nodes.

Variables such as patient age, gender, clinical and pathological staging, final histology, grading, type of treatment modality used, the extent of neck dissection, margin status, and site of recurrence were recorded and evaluated. We have grouped patients into early (T1-T2) and locally advanced (T3-T4) disease stages to avoid dispersing the data. Patients were also categorized into clinically node negative and positive as well as pathologically node negative and positive groups to analyze survival.

Data were analyzed using IBM SPSS Statistics for Windows, Version 20.0 (released 2011, IBM Corp. Armonk, NY). Kaplan Meier curves were used to analyze survival data.

The study was reviewed by the Institutional Review Board, and the exemption was granted by the Ethical Review Committee of Shaukat Khanum Memorial Cancer Hospital and Research Center, Lahore, Pakistan.

## Results

We retrospectively analyzed the medical records of 209 patients diagnosed with malignant parotid neoplasms. There were 119 men and 94 women. One hundred sixty-four patients were primarily treated with surgery or surgery followed by radiotherapy in our hospital while 39 patients underwent operations in a different facility but were given adjuvant treatment in our clinic. The median patient age at diagnosis was 40 years. The median follow-up was 24 months. Table 1 shows the clinical and pathological staging, histology, type of parotidectomy, and treatment modality used. Clinical tumor stage showed cT1 (49 cases), cT2 (65 cases), cT3 (31 cases), and cT4 (64 cases). Pathological classification showed pT1 (43 cases), pT2 (60 cases), pT3 (38 cases), and pT4 (23 cases).

Eighty-two patients received superficial parotidectomy, 71 patients received complete parotidectomy, nine patients received radical parotidectomy, and two patients received extended radical parotidectomy.

Forty-one patients received neck dissection as part of the procedure, and 36 of those were classified as pN+ while five were classified pN0.

Histology revealed mucoepidermoid carcinoma to be the most common malignancy (with a 50% incidence rate) followed by adenoid cystic carcinoma (10.5%) and adenocarcinoma (9.6%). Only 23 patients received surgery alone while 141 patients received surgery followed by radiotherapy. Thirty-nine patients underwent operations in a different facility, and these patients received postoperative radiotherapy in the presence of adverse conditions such as a close/positive margin, high-grade malignancy, tumor size >4 cm, facial nerve involvement, and extra-parenchymal extension. The median postoperative radiotherapy dose was 60 Gy on the parotid gland and 55 Gy on the neck. Adjuvant chemotherapy was used in select patients who had gross lymph nodal involvement.

**Figure 1:**
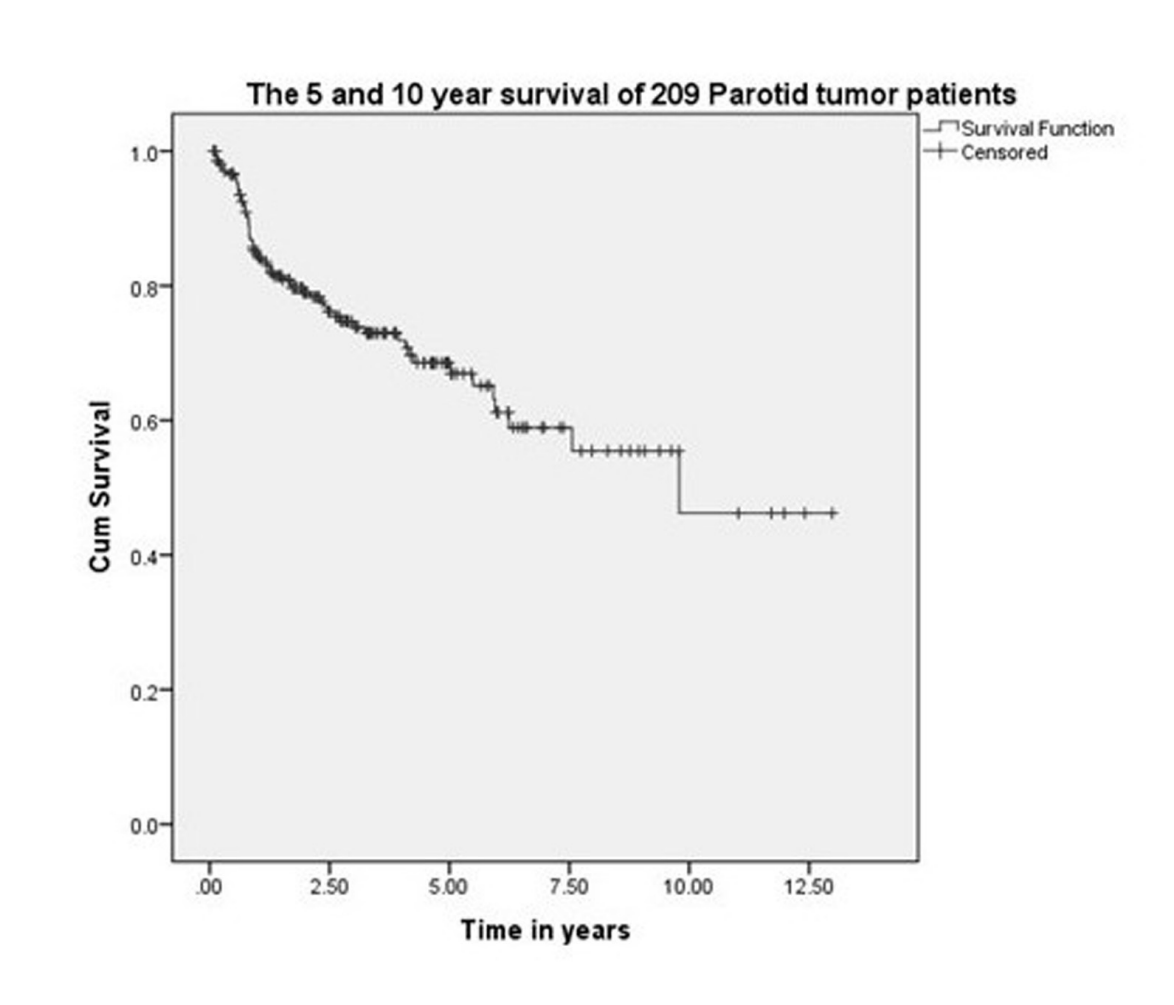
5- and 10-year survival of 209 malignant parotid tumor patients.

**Figure 2:**
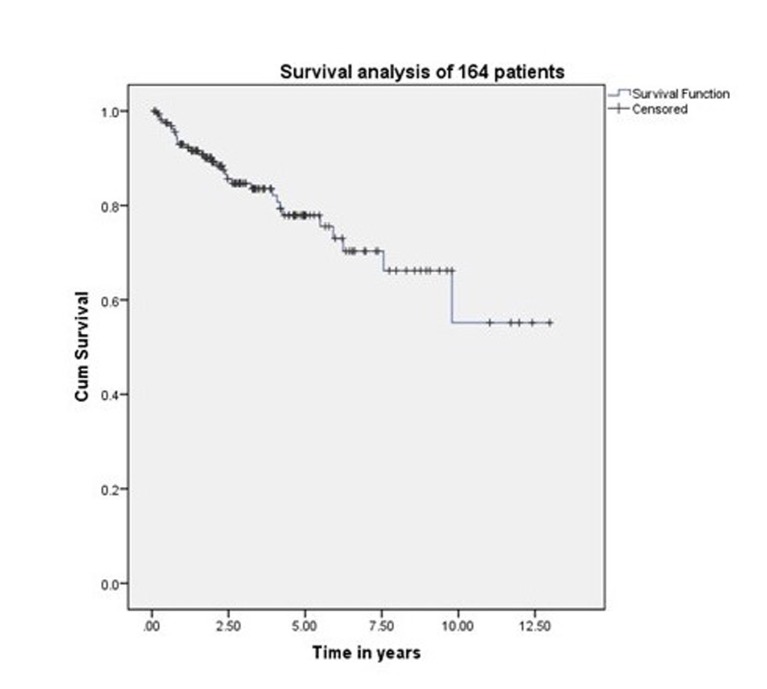
Survival of 164 patients treated with surgery and radiotherapy.

**Figure 3:**
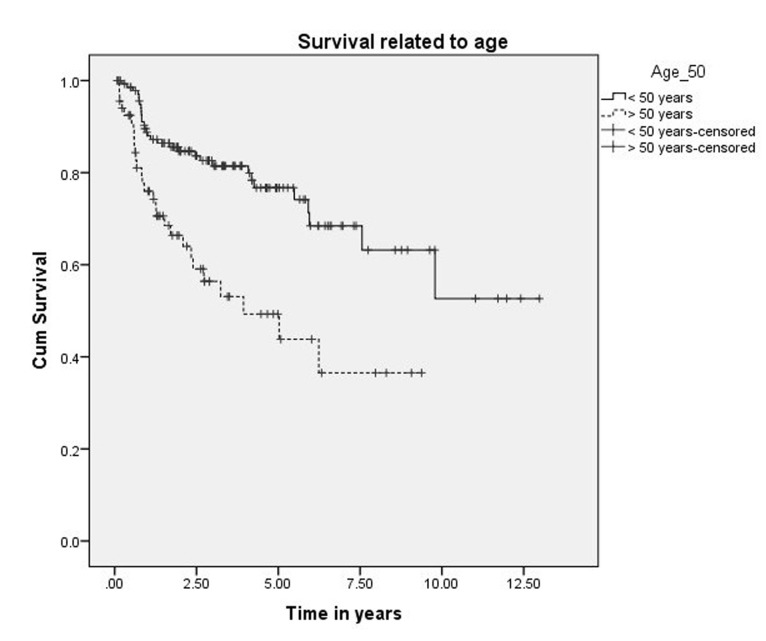
Survival related to age.

**Figure 4:**
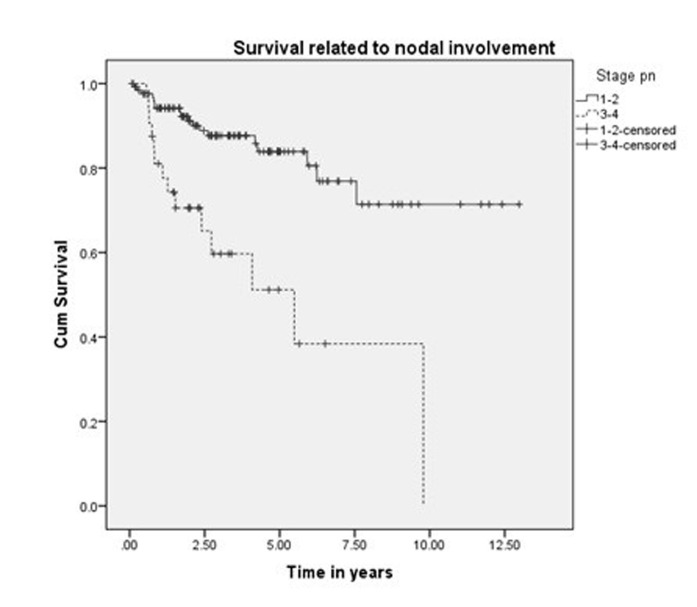
Survival related to nodal stage.

**Figure 5:**
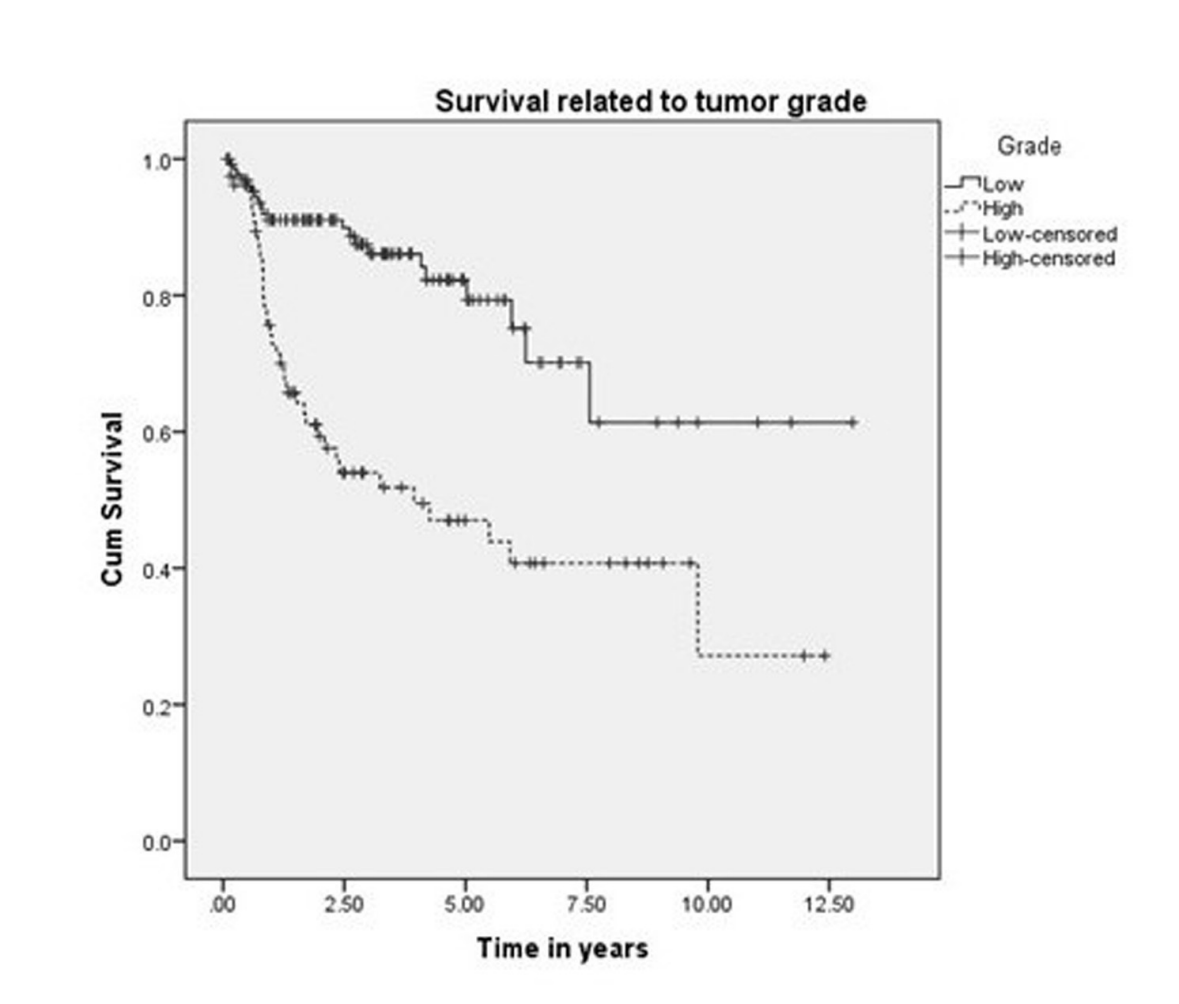
Survival related to grade.

**Figure 6:**
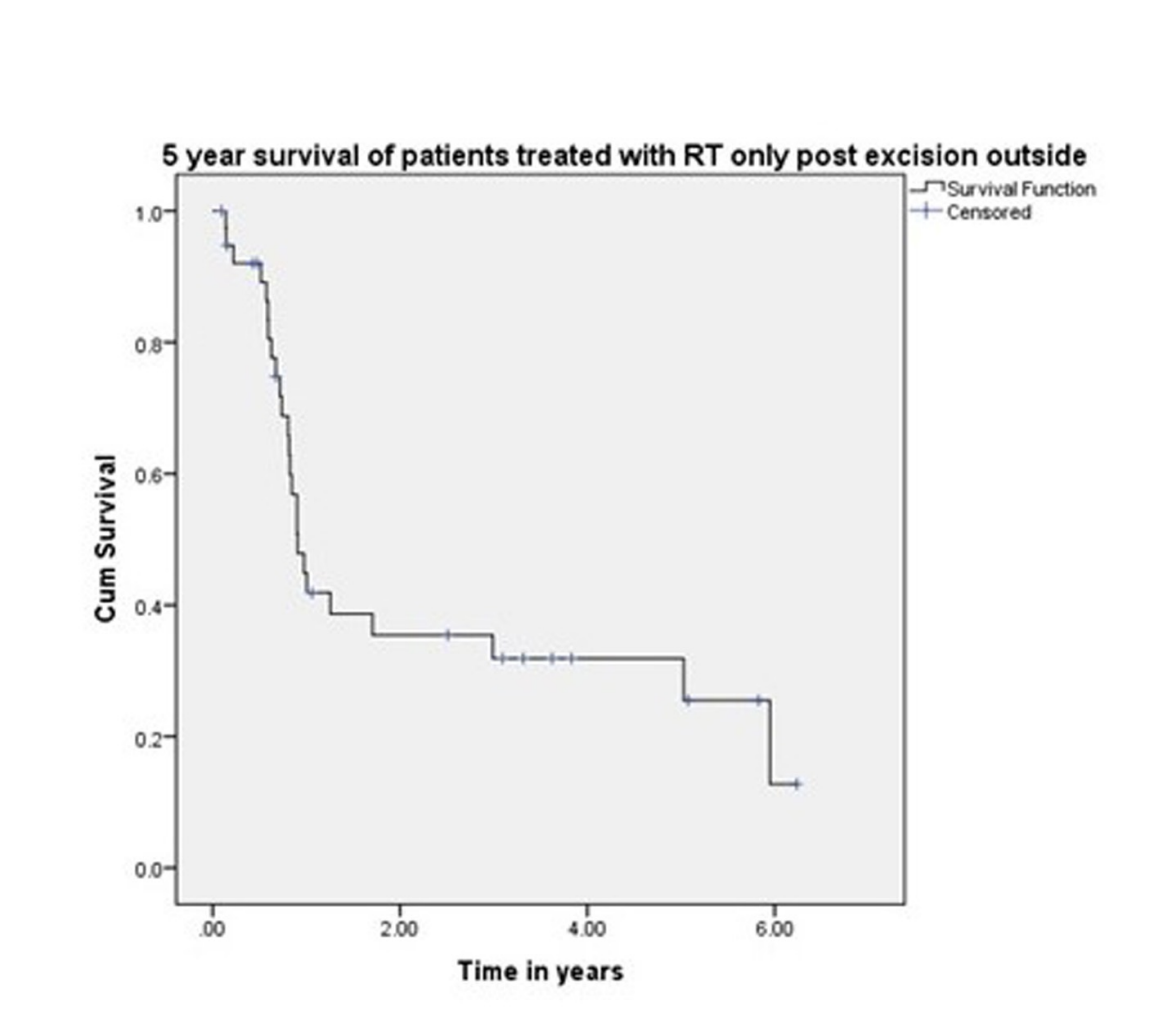
Survival of patients treated outside.

The five-year and 10-year overall survival was 68% and 45%, respectively, for all the patients (Figure 1). The five-year and 10-year survival rates were 78% and 54%, respectively, for those treated exclusively in our hospital (Figure 2). Variables, which have statistical significance on survival, were age (categorized as either <50 or >50 years; p = 0.029), grade (high vs low; p < .001), pathological nodal involvement (pN0 vs pN+; p < .001), and pathological tumor size (pT1-2 vs pT3-4; p < 0.001) as shown in Table 2 and Figures 3-5. Multivariate analysis showed age above 50 years, high grading, advanced pathological stage, and nodal involvement to be significant factors affecting survival (Table 3). The 5-year survival of patients who were previously treated outside our hospital has not shown promising results in terms of survival (Figure 6).

**Table 1:** Clinico-pathological characteristics.

	N	%
Age		
<50 years	141	67.5
>50 years	68	32.5
Sex		
Male	115	55
Female	94	45
cT		
1	49	23.4
2	65	31.1
3	31	14.8
4	64	30.6
pT		
1	43	26.2
2	60	36.5
3	38	23.1
4	23	14
cN0	156	74.6
cN+	53	25.3
pN0	128	78
pN+	36	22
Surgical treatment type		
Superficial parotidectomy	82	50
Completion parotidectomy	71	43.2
Radical parotidectomy	9	5.4
Extended radical parotidectomy	2	1.2
Histology		
Mucoepidermoid carcinoma	105	50.2
Adenoid cystic carcinoma	27	12.9
Adenocarcinoma	22	10.5
Squamous cell carcinoma	13	6.2
Acinic cell carcinoma	11	5.2
Carcinoma Ex-pleomorphic adenoma	9	4.3
Others	22	10.5
Treatment modality		
Surgery	23	11
Surgery + Adjuvant radiotherapy	141	67.4
Adjuvant radiotherapy	39	18.6
Adjuvant chemotherapy	6	2.8
Neck dissection		
Yes	41	19.6
No	168	80.3

**Table 2:** Prognostic factors affecting survival outcome. ND: Neck dissection; cN0: Clinically negative neck nodes; cN+: Clinically positive/palpable nodes neck; pN0: Pathologically negative neck nodes; pN+: Pathologically positive neck nodes; PNI: Perineural invasion; PORT: Post operative radiotherapy.

	5-Year Survival	10-Year Survival	p-value
Age < 50 years	82%	58%	.029
>50 years	62%	51%	
Male	77%	51%	0.57
Female	77%	61%	
c T1-T2	86%	79%	<0.0001
c T3-T4	43%	14%	
p T1-T2	88%	71%	<0.0001
p T3-T4	58%	25%	
c N0	77%	65%	<0.0001
c N+	33%	0%	
p N0	85%	73%	<0.0001
p N+	55%	0%	
ND: Yes	74%	74%	<0.0001
ND: No	85%	79%	
Grade			
High	56	30	<0.001
Low	87	70	
PNI			
Yes	56%	0%	0.08
No	77%	58%	
Extra-parenchymal extension			0.150
Yes	66%	0%	
No	75%	54%	
Adjuvant PORT			0.250
Yes	76%	49%	
No	90%	90%	

**Table 3:** Multivariate analysis affecting survival outcome.

Variable	p-value	Hazard Ratio
Age (<50 years vs >50 years)	0.127	1.8
Grade (Low vs High)	0.05	2.1
pT1-2 vs pT3-4	0.02	2.6
pN0 vs pN+	0.01	2.7

## Discussion

Most salivary neoplasms originate in the parotid gland, while minor salivary gland and submandibular gland neoplasms are much less common. A low incidence combined with histological heterogeneity poses a challenge in evaluating survival and oncological outcomes [9].

Age and gender have been postulated as prognostic factors in studies published by Malata, et al., Pohar, et al., Koul, et al., Jouzdani, et al., and Lima, et al., in contrary to a study by Mercante, et al. reporting no difference in survival [10-15]. Based on our findings, age is an important prognostic factor in terms of survival (p = 0.029), but no significant difference was found as far as gender is concerned (Figure 3).

Harbo, et al. established a relationship related to tumor stage and histological classification [16]. A retrospective study on 152 patients with parotid tumors found a statistically significant difference in survival based on tumor stage. The survival rate for Stage I and II tumors was 65% and 50%, respectively, and falls to 9% in Stage IV patients [17]. The prognosis for survival in histologically well-differentiated tumors (52%) was also better than the prognosis associated with poorly differentiated lesions (19%). Our experience showed that survival related to malignant parotid tumors depends upon cT (p < 0.0001), pT (p < 0.0001), cN (p < 0.0001), pN (p < 0.0001), histology (p < 0.001), and ND (p < 0.0001), which is comparable to other retrospective series as demonstrated by Lima, et al., Terhaard, et al., and Stenner, et al. [14,17,18]. Thus, patients with clinical and pathological tumor sizes >T2, high-grade histology, clinical and pathological nodal involvement, and perineural involvement have worse five-year and 10-year survival (Figure 4 and Figure 5). The role of adjuvant radiotherapy is reported to improve survival according to Armstrong, et al. and Mendenhall, et al. [19-20], but our results indicated no such benefit (Figure 2). Postoperative radiation therapy may reduce the risk of loco-regional failure, thus decreasing the risk of damage to the facial nerve in cases of loco-regional failures. Clear evidence of a dose-response is lacking in this rare disease, and the dose of 60 Gy in daily 2 Gy fractions represents a practical consensus. Developments in radiotherapy technique such as intensity modulated radiotherapy have been applied to parotid treatment [21,22]. A careful analysis of our data has shown that patients with adverse features were candidates for adjuvant radiotherapy, which may explain the poor survival outcome in these individuals. As the only tertiary care referral cancer hospital, we cannot refuse patients who have been previously treated elsewhere with inadequate resections. Of 209 malignant parotid tumor patients, 39 had been accepted for adjuvant treatment after surgery in a facility outside of our hospital. The five-year survival of these individuals was only 25% (Figure 6). In our experience, this poor outcome is mostly attributed to open biopsies outside contaminating the surrounding bed by spillage of tumor cells, fragmented nature of the resected specimen, delayed presentations for adjuvant treatment post-primary surgery and attempting inoperable tumors leaving behind gross residual disease by outside surgeons. Similarly, the role of extraparenchymal extension affecting survival has been described by Klussmann, et al., but its role is not statistically evident according to our results. This may be due to only a small number of patients having extraparenchymal extension, and larger sample size may highlight its role in survival [23].

## Conclusions

We evaluated oncological outcomes of malignant parotid salivary gland tumors by identifying the prognostic factors affecting survival. Advanced age, high-grade histology, increased size (>T2) and clinicopathological nodal involvement adversely affect overall survival. Adjuvant radiotherapy, however, has not resulted in improved survival particularly in the background of advanced disease.
